# Editorial: The role of essential trace elements in health and disease

**DOI:** 10.3389/fpubh.2023.1285603

**Published:** 2024-01-03

**Authors:** Dong-Xing Guan, Chunguang Yang, Jerome O. Nriagu

**Affiliations:** ^1^Institute of Soil and Water Resources and Environmental Science, College of Environmental and Resource Sciences, Zhejiang University, Hangzhou, China; ^2^Department of Urology, Tongji Hospital, Tongji Medical College, Huazhong University of Science and Technology, Wuhan, China; ^3^Department of Environmental Health Sciences, School of Public Health, University of Michigan, Ann Arbor, MI, United States

**Keywords:** essential trace elements, human health, metabolic diseases, maternal and perinatal health, human cancers, public health

Research during the last half century has clearly established that trace elements, whether essential or non-essential, play important roles in a wide variety of biological processes of living systems and can also be a defining factor in the outcome of parasitic infections (1). Until recent years, most of the public health attention and funding were directed at the worldwide contamination of the environment with toxic but non-essential trace metals, especially the so-called “big four” of arsenic, cadmium, lead, and mercury. The bioaccumulation and biomagnification of these heavy metals oftentimes increase their potential for toxicity further up the food chain, thereby increasing the level of the public's concern. Anthropogenic trace elements are non-degradable and are deemed to pose a continuous risk for human and animal health for a long time. Since the sources of pollution are well-defined, they are amenable to study and remediation.

In recent decades, attention is increasingly being focused on the essential trace elements (often called micronutrients) that occur naturally in small amounts and play critical roles in numerous physiological and metabolic processes in both plants and animals. The role of essential trace metals in health and disease is enigmatic considering that their exposure-disease relationships have evolved over time to be U-shaped mostly. Very low levels of an essential trace element in biological systems give rise to symptoms of deficiency. This is followed by a range in tissue concentrations where an organism is able to maintain biological functions at the optimum level of the trace element. Finally, at some higher tissue level of the trace element, the normal regulatory mechanisms are overloaded, resulting in toxic symptoms. Each element has its own characteristic exposure-response curve while the optimum concentration range can differ by as much as several orders of magnitude depending on the chemical form of the element and the specific compound in the exposure dose. Most toxicological studies assume simple linear exposure-response relationships and ignore the fact that exposures in the natural environment oftentimes fall outside the optimum concentrations to maintain physiological and metabolic processes. Failure to account for the U-shaped form of the exposure-disease relationships is believed to be responsible for much of the confusing and conflicting results that have been published on the interconnection of trace elements with human health.

This Research Topic is not intended to be a comprehensive coverage of the huge volume of literature on the diverse roles that essential trace elements play in human health and disease. Rather, the articles have been selected to illustrate areas of emerging interests within the broad field. The papers cluster into three broad themes or public health problems of global concern, namely, the role of trace elements in metabolic diseases; maternal and perinatal health; and human cancers.

Trace elements have been identified for long time as potential candidates for improving metabolic disorders including insulin resistance, obesity, metabolic syndrome, and diabetes. Studies of the past few years clearly show that micronutrient nutrition is one of the most important modifiable lifestyle factors for preventing disease and maintaining health. As a consequence, this topic has been an active field of investigation for a long time.

Wu et al. used the Guangdong Provincial Residents' Chronic Disease and Nutrition Surveillance Survey data (2015) to extend the understanding of the relationship between exposure to multiple metals and hypertension. They found that higher levels of manganese, zinc and selenium are associated with increased risks for hypertension and elevated blood pressure readings in the general population of southern China. Wu et al. found synergistic interactions of the essential micronutrients with the non-essential toxic elements (arsenic, cadmium, and lead) in the mediation of hypertension by obesity. This finding is important since essential and non-essential trace elements tend to co-occur in many environmental media. Evidence for the association of obesity with levels of lead and cadmium in blood was found to be weak in this report. In another multiple-metal study, González-Domínguez et al. explored the role of dietary trace elements on the etiology of obesity and related disorders. They found that alterations in concentrations of trace elements in peripheral blood were closely correlated with the characteristic metabolic complications behind childhood obesity, namely hyperglycemia, hyperinsulinemia, and dyslipidemia in a cohort of Spanish children and adolescents.

The effects of specific trace elements alone on metabolic disorders are discussed in several articles. The paper by Ren et al. contributes to the growing fund of knowledge on the relationships between iron markers (including ferritin, transferrin, and soluble transferrin receptor) and metabolic obesity phenotypes. Non-alcoholic fatty liver disease (NAFLD) is a multistage condition that affects 30% of the global population, and is causally linked to end-stage liver disease. Growing evidence indicates that NAFLD is the hepatic manifestation of metabolic syndrome—obesity being a well-known predisposing factor for NAFLD (Zhu et al.). The article by Zhu et al. contributes to the growing body of evidences that increasingly show that dietary fiber intake from plant foods or supplements could confer a greater benefit in reducing the NAFLD risk and improving liver function. The role of trace elements in the association of obesity with NAFLD remains equivocal according to a meta-analysis of pooled data of 2,607 NAFLD patients and 1,441 non-NAFLD normal individuals. This review concluded that there was no significant association between serum copper or ceruloplasmin with NAFLD, even although NAFLD patients had low hepatic copper concentrations (Chen Y. et al.).

The effects of trace elements on maternal and perinatal health have been a matter of public health interest for a long time. One topic in this area that is receiving increased attention is preeclampsia, a leading cause of maternal and perinatal mortality especially in the low- and middle-income countries. Disruptions in metabolic cycles of trace elements are suspected as playing a vital role in developing preeclampsia. One of the metal suspects is copper. In pregnancy, aberrant maternal copper levels may give rise to early spontaneous miscarriage, fetal structural anomalies, gestational diabetes, small-for-gestation babies, and preterm birth—hallmarks of preeclampsia. From their systematic review of the published literature, Zhong, Yang, Sun et al. concluded that disruptions in maternal copper levels are correlated with risks of preeclampsia, but the resulting pathologies present variously across different geographical and economic contexts. Equally important, the experimental protocols used in most studies made it impossible to relate the exposure dose to the bounding concentrations for copper deficiency vs. copper toxicity. Similar conclusions were reached in a review and meta-analysis of maternal serum zinc level as a predisposing factor for preeclampsia (Jin et al.). By contrast, a meta-analysis of pooled data from 21 studies in different countries showed that that maternal lead exposure is unequivocally associated with preeclampsia during pregnancy, even at very low levels (Zhong, Yang, Li et al.).

A large volume of literature exists to show that trace elements play critical roles, both good and bad, in human carcinogenesis but the underlying mechanisms are still not well understood. The discovery of the so-called “esophageal cancer belts” in South Africa, France, Iran, and China has recently generated a lot interest on the etiology of esophageal cancer, the seventh leading cause of cancer death worldwide. Smoking, alcohol consumption, exposure to environmental pollutants and diet Are the generally recognized risk factors for esophageal carcinoma. Strong associations with exposure to trace elements have been reported but the results are not coherent. The timely article by Yang et al. provides a good overview of the connections between zinc, copper, iron, and selenium and esophagus cancer with emphasis on the likely underlying mechanisms.

Individual papers in this Research Topic offer comprehensive reviews of current knowledge or present the results of cutting-edge research. The articles come from a range of disciplines and hopefully should add to the growing effort to integrate human health and ecosystem health under the emerging field of One Health. The papers together make it clear that a better understanding of the roles of the essential trace elements would be instrumental in the development of new dietary intervention measures to improve global health. We thank the authors of the articles who deserve the credit for the quality of this Research Topic. We also express our sincere appreciation to the reviewers who contributed immeasurably to the betterment of the papers.

## Author contributions

D-XG: Writing—original draft, Writing—review & editing. CY: Writing—original draft. JN: Writing—original draft, Writing—review & editing.
